# Patient- vs organ-based prognostic tools for older patients in critical care units: An observational study with a 3-month follow-up

**DOI:** 10.1007/s00063-024-01179-z

**Published:** 2024-09-25

**Authors:** Melanie Hochleitner, Lena Pickert, Nick A. Nolting, Anna Maria Affeldt, Ingrid Becker, Thomas Benzing, Matthias Kochanek, Maria Cristina Polidori

**Affiliations:** 1 Ageing Clinical Research, Department II of Internal Medicine and Center for Molecular Medicine Cologne, University of Cologne, Faculty of Medicine and University Hospital Cologne, Cologne, Germany; 2 Institute of Medical Statistics and Computational Biology, University of Cologne, Faculty of Medicine and University Hospital Cologne, Cologne, Germany; 3 Cologne Excellence Cluster on Cellular Stress Responses in Aging-Associated Diseases (CECAD), University of Cologne, Faculty of Medicine and University Hospital Cologne, Cologne, Germany; 4 First Department of Internal Medicine, Faculty of Medicine and University Hospital Cologne, Center of Integrated Oncology Aachen Bonn Cologne Düsseldorf, University of Cologne, University Hospital Cologne, Cologne, Germany

**Keywords:** Frailty, Multidimensional Prognostic Index (MPI), Aging medicine, Intensive care medicine, Mortality risk, Gebrechlichkeit, Multidimensionaler prognostischer Index (MPI), Altersmedizin, Intensivmedizin, Letalitätsrisiko

## Abstract

**Background:**

Anticipating a doubling of older adults in Europe by 2050, healthcare systems face substantial challenges, particularly in critical care units. However, there is still a lack of evidence-based knowledge for treating and assessing mortality risk in older patients. This study compared the predictive accuracy of two assessment tools for long-term outcomes among older patients: the Multidimensional Prognostic Index (MPI) and the Sequential Organ Failure Assessment (SOFA). As the MPI is based on a more holistic assessment, it may provide a more accurate prediction than the organ-based SOFA.

**Objective:**

Does the MPI provide a more accurate prediction of mortality risk and quality of life for older patients in critical care units than the organ-based SOFA score?

**Methods:**

In a 6-month study, 96 patients aged 65 and older admitted to intensive (ICU) or intermediate care units (IMC) were enrolled to assess 90-day mortality using a comprehensive geriatric assessment-based MPI and the SOFA score. The follow-up (FU) involved telephone assessments 30 and 90 days after admission, focusing on posthospitalization health and quality of life.

**Results:**

Both MPI (*p* = 0.039) and SOFA score (*p* = 0.014) successfully predicted mortality among older IMC and ICU patients in logistic regressions. Receiver operating characteristic (ROC) analyses demonstrated comparable areas under the curve (AUCs) for MPI (0.618) and SOFA score (0.621), as well as a similar sensitivity and specificity (MPI 61.0% and 52.9%; SOFA score: 68.9% and 45.1%, respectively). The MPI at admission moreover correlated significantly with quality of life (*p* < 0.001, r = −0.631 at discharge; *p* = 0.005, r = −0.377 at 30-day FU; *p* = 0.004, r = −0.409 at 90-day FU) and nursing needs (Mann–Whitney U‑test, *p* = 0.002 at 30-day FU; *p* = 0.011 at 90-day FU) at FU, while the SOFA score did not show significant associations with respect to these parameters.

**Conclusions:**

In geriatric critical care, both the MPI and the SOFA score effectively predict mortality risk. While the SOFA score may appear more practical due to its simpler and faster implementation, only the MPI demonstrated significant correlations with quality of life and nursing needs in the FU after 30 and 90 days.

## Abstract (de)

**Hintergrund:**

Bis 2050 wird eine Verdopplung älterer Patient:innen in Europa erwartet, was Gesundheitssysteme, insbesondere Intensivstationen, vor große Herausforderungen stellt. Zudem fehlt es an evidenzbasiertem Wissen für die Behandlung und Einschätzung des Letalitätsrisikos älterer Patient:innen. Diese Studie verglich die Vorhersagepräzision zweier Scores für die langfristige Beurteilung des Letalitätsrisikos und der Lebensqualität bei älteren Patient:innen: Multidimensional Prognostic Index (MPI) und Sequential Organ Failure Assessment (SOFA). Da der MPI auf einer umfangreicheren Beurteilung basiert, könnte dieser eine präzisere Vorhersage ermöglichen als der organbasierte SOFA-Score.

**Ziel:**

Kann der MPI im Vergleich zum organbasierten SOFA-Score das Letalitätsrisiko und den Einfluss auf die Lebensqualität bei älteren Intensivpatient:innen präziser vorhersagen?

**Methoden:**

In einer 6‑monatigen Studie wurden 96 Patient:innen ≥ 65 Jahren auf Intensiv- (ICU) und Intermediate Care (IMC) Stationen erfasst. Das 90-Tage-Letalitätsrisiko wurde mittels MPI und SOFA-Score ermittelt. Anschließend erfolgte 30 und 90 Tage nach initialem Assessment ein telefonisches Follow-up (FU) mit Fokus auf die Lebensqualität und den Gesundheitszustand nach Hospitalisierung.

**Ergebnisse:**

Sowohl der MPI (*p* = 0,039) als auch der SOFA-Score (*p* = 0,014) zeigten eine gute Vorhersagekraft für Letalität bei älteren Intensivpatient:innen in logistischen Regressionen. Eine Receiver Operating Characteristic (ROC) Analyse zeigte vergleichbare „areas under the curve“ (AUC) für MPI (0,618) und SOFA-Score (0,621) sowie ähnliche Werte für Sensitivität und Spezifität (MPI: 61,0 % bzw. 52,9 %; SOFA-Score: 68,9 % bzw. 45,1 %). Der MPI bei Aufnahme zeigte zudem eine signifikante Korrelation mit der Lebensqualität (*p* < 0,001, r = −0,631 bei Entlassung, *p* = 0,005, r = −0,377 nach 30 Tagen FU; *p* = 0,004, r = −0,409 nach 90 Tagen FU) und dem Pflegegrad (Mann-Whitney-U-Test, *p* = 0,002 nach 30 Tagen FU; *p* = 0,011 nach 90 Tagen FU). Für den SOFA-Score ergaben sich bei diesen Parametern keine signifikanten Ergebnisse.

**Schlussfolgerung:**

In der geriatrischen Intensivmedizin können sowohl der MPI als auch der SOFA-Score das Letalitätsrisiko effektiv vorhersagen. Während der SOFA-Score aufgrund seiner einfacheren und schnelleren Anwendung praktischer erscheinen mag, zeigte nur der MPI bei den FUs nach 30 und 90 Tagen signifikante Korrelationen mit Lebensqualität und Pflegegrad.

## Background

According to projections, the number of individuals in Europe aged above 80 is expected to double by 2050 [31]. The consequent rise in multimorbidity, frailty, and chronic diseases is placing increasing pressure on intensive (ICU) and intermediate care units (IMC) [1, 6]. Understanding challenges linked to this demographic shift is crucial for contemporary societies and healthcare systems [20]. Despite these trends, there is a significant lack of evidence-based knowledge regarding the treatment of older adults, who are often excluded from randomized controlled trials [9]. This knowledge gap raises concerns, particularly given the pivotal role of early frailty recognition—a marker for biological age—in shaping medical care for older patients. Such recognition not only facilitates a more accurate assessment of mortality risk but also positively influences patient outcomes [28]. One precise frailty assessment tool is the Multidimensional Prognostic Index (MPI), based on a comprehensive geriatric assessment (CGA) [36]. Despite its commendable clinimetric properties and successful prognostications across various outcomes, including mortality and rehospitalizations [16, 25], it has rarely been used in critical care units. Another well-established tool for predicting mortality risk, especially in intensive care medicine, is the Sequential Organ Failure Assessment (SOFA) [33]. This study aimed to compare the predictive power of the MPI [24] and of the SOFA for older patients in critical care units not only in terms of mortality risk, but also with respect to quality of life and nursing needs.

## Patients and methods

Over a 6-month daily recruitment period, patients (≥ 65 years) admitted to ICU or IMC were enrolled within 48 h. Exclusion criteria were a lack of German language skills, machine-invasive ventilation, immediate life-threatening conditions, or a stay > 48 h in the respective ward before screening. Main endpoint of this study was mortality at 90-day follow-up (FU).

### Clinical evaluation

The key assessments were a CGA-based calculation of the MPI as well as the SOFA. Moreover, 17 geriatric syndromes (GS) and 10 geriatric resources (GR) were collected. The GS include incontinence, instability, cognitive alteration, depression or irritability, inanition, sensory impairment, chronic pain, insomnia, irritable bowel syndrome, impoverishment, isolation, immobility, polypharmacy, iatrogenic disease, incoherence/delirium, fluid/electrolyte imbalance, and swallowing disorders. GR comprise favorable intellectual, physical, social, and economic resources, good living conditions, and motivational, emotional, mnemonic, and competence-related resources [19]. Subsequent to recruitment and obtaining consent, study participants underwent a FU visit by phone 30 and 90 days after initial evaluation. Information on survival, admission to long-term care facilities, nursing needs (German: *Pflegegrad*), GP/outpatient visits, rehospitalizations, drug prescription, falls, and quality of life by means of the European Quality of Life‑5 Dimensions (EQ-5D-5L) scale [10] was collected at discharge and FU. The EQ-5D-5L consists of five dimensions: mobility, self-care, usual activities, pain/discomfort, and anxiety/depression. Each dimension is evaluated on a 5-point scale, ranging from 1 to 5, with 1 indicating no issues and 5 indicating severe impairment. The scores of these individual dimensions can be transformed into a unified index score, ranging from −0.661 to 1, using population-based preference weights. Higher index values, thereby, correspond to greater levels of quality of life [32].

### MPI

The MPI comprises eight subscales, including Activities of Daily Living (ADL) [13], Instrumental Activities of Daily Living (IADL) [15], Cumulative Illness Rating Scale—Comorbidity Index (CIRS) [17], Mini Nutritional Assessment Short Form (MNA-SF) [30], Short Portable Mental Status Questionnaire (SPMSQ) [21], Exton Smith Scale (ESS) [3], number of medications, and social housing situation [24]. The resulting continuous MPI value ranges from 0 to 1, with higher values indicating a higher risk. This allows a classification into three risk groups: MPI‑1 (0.0–0.33), MPI‑2 (0.34–0.66), and MPI‑3 (0.67–1.0) [24]. Due to a limited number of patients in the low-risk MPI‑1 group (*N* = 6), a combined low- and medium-risk group was formed for this analysis.

### SOFA

The SOFA score evaluates six organ systems (respiratory, cardiovascular, hepatic, coagulation, renal, and neurological) with a separate score from 0 to 4. For instance, liver function is determined by bilirubin levels, with scores ranging from 0 to 4. The cumulative score is then calculated by adding up the individual dimensions and ranges from 0–24. Higher values reflect a higher mortality risk and organ dysfunction [33]. SOFA scores were computed using pre-existing participant data in this study.

### Registration, participant consent, and ethics

This is a prospective observational study, conducted by the Ageing Clinical Research team at the University Hospital Cologne, collaborating with the Department I for Oncology, Hematology, and Internal Intensive Medicine. Ethical approval was granted by the Cologne Ethics Committee (EK19-1049_1, 18 June 2019), and the study was registered in the German Clinical Trials Registry (DRKS00016951). Informed consent was obtained from all patients or their authorized representatives.

### Statistical analysis

Statistical analyses were performed using SPSS Statistics (Version 26.0; IBM, Armonk, NY, USA). Descriptive statistics included absolute numbers and relative frequencies for categorical variables and medians with interquartile ranges (IQR) for continuous variables. Given the nonnormal distribution of all variables (Shapiro–Wilk tests), nonparametric methods were employed to compare continuous variables between groups. Rates were compared using Χ^2^ or Fisher’s exact test. Logistic regression analyses reported as average marginal effects (ME) and adjusted for age and gender were conducted to evaluate the predictive power of MPI and SOFA score on mortality and nursing needs. MEs indicate how the probability of an event changes on average when an independent variable is increased by one unit. They are particularly useful because they allow a more intuitive interpretation of the results of a logistic regression by quantifying the change in probability rather than just looking at the log-odds. Additionally, a receiver operating characteristic (ROC) analysis was employed to compare both scores for mortality risk. Pearson correlation analyses were performed to examine the correlation between MPI and SOFA score with quality of life at both FUs. Ordinary least squares (OLS) regressions were used to check for robustness and control for age and gender.

## Results

### Study population and demographics

**Fig. 1:**
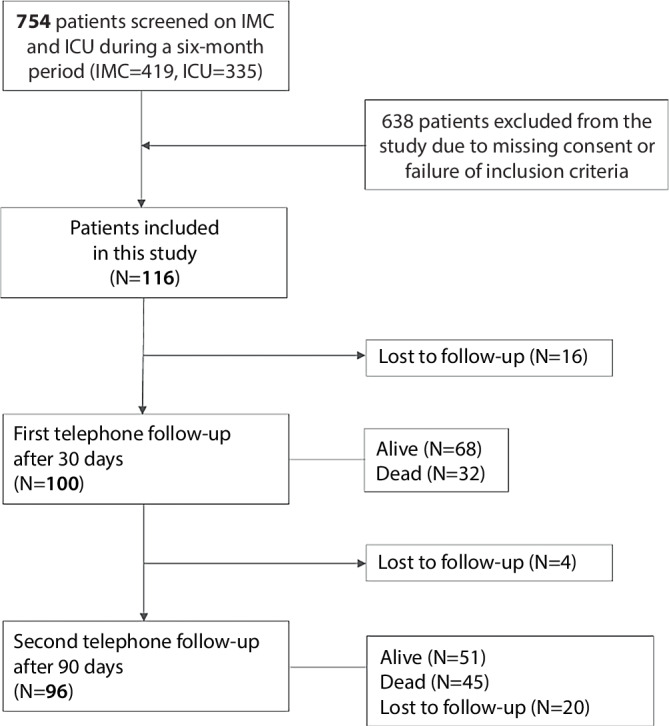
Flow chart of the study

Figure 1 depicts the stepwise inclusion and exclusion of patients, yielding a final sample size of 96. Detailed demographics and clinical characteristics, including MPI and SOFA subgroups, are presented in Table 1. The median age was 74 (44% women). Of all patients, 81.3% were admitted to IMC and 18.7% to ICU. After 3 months, 46.9% had died. At admission, the median MPI was 0.69 (IQR = 0.56–0.75), and the median SOFA score was 4 (IQR = 2–7). The median hospital stay was 25 days, with a daily medication count of 9 (IQR = 7–12). Common admission reasons included infections (30.2%), malignancies (17.7%), and electrolyte disorders (12.5%).

**Table 1:** Study population

	Total (*N* = 96)	Alive (*N* = 51)	Deceased (*N* = 45)	*p*-value^a^
*Demographics*				
Age (years), median (IQR)	74 (69–80)	74 (69–80)	75 (69–80)	0.627
Female, *N* (%)	44 (45.8)	23 (45.1)	21 (46.7)	0.878
IMC, *N* (%)	78 (81.3)	44(86.3)	34(75.6)	0.179
ICU, *N* (%)	18 (18.7)	7 (13.7)	11 (24.4)	0.179
LHS, median (IQR)	25 (8–44)	18 (8–40)	30 (12–47)	0.220
*MPI groups, N* (%)	–	–	–	0.087
MPI‑1 & MPI‑2	43 (44.8)	27 (52.9)	16 (35.6)	–
MPI‑3	53 (55.2)	24 (47.1)	29 (64.4)	–
MPI value, median (IQR)	0.69 (0.56–0.75)	0.63 (0.50–0.69)	0.69 (0.56–0.78)	0.051
*MPI domains, median (IQR)*				
CIRS	4 (3–6)	4 (3–5)	5(3–6)	0.106
ADL	1(0–3)	2(1–6)	1(0–2)	*0.005*
IADL	2(1–3)	2(1–3)	1(0–3)	*0.022*
MNA-SF	7(5–10)	7(5–10)	7(4–9)	0.238
SPMSQ	2(1–4)	1(1–3)	3(1–5)	*0.004*
ESS	13 (10–15)	14(11–17)	12(9–14)	*0.005*
Number of medications	9 (7–12)	8 (7–12)	9 (7-13)	0.338
Living conditions, *N* (%)	–	–	–	0.833
– With relatives	65 (68.4)	34 (66.7)	31(68.9)	–
– Institutionalized/private attendant	6 (6.3)	4 (7.8)	2 (4.4)	–
– Alone	24 (25.3)	13 (25.5)	11 (68.9)	–
*SOFA score domains, median (IQR)*				
Glasgow coma scale	15 (14–15)	15 (15–15)	15 (14–15)	0.277
PaO_2_/FiO_2_	362 (281–434)	362 (267–433)	395 (298–478)	0.052
Mean arterial pressure	80 (70–89)	81 (73–93)	77 (63–87)	*0.027*
Vasopressors, *N* (%)	16 (16.7)	5 (9.8)	11 (24.4)	0.055
Thrombocytes	134 (60–240)	167 (70–243)	115 (55–230)	0.223
Creatinine	1.1 (0.8–2.3)	1.0 (0.8–1.6)	1.3 (0.8–3.0)	0.432
Bilirubin	0.6 (0.4–1.1)	0.5 (0.3–0.9)	0.7 (0.4–2.0)	0.116
SOFA score, median (IQR*)*	4 (2–7)	4 (2–5)	5 (3–7)	*0.040*

*IQR* Interquartile range, *IMC* Intermediate care, *ICU* Intensive care unit, *LHS* Length of hospital stay, *MPI* Multidimensional Prognostic Index, *CIRS* Cumulative Illness Rating Scale, *ADL* Activities of Daily Living*, IADL* Instrumental Activities of Daily Living*, MNA-SF* Mini Nutritional Assessment Short Form, *SPMSQ* Short Portable Mental Status Questionnaire, *ESS* Exton Smith Scale, *SOFA* Sequential Organ Failure Assessment, *PaO*_*2*_*/FiO*_*2*_ Oxygen partial pressure to the inspiratory oxygen concentration.

^a^ *P*-values based on non-parametric methods or Χ^2^/Fisher’s exact tests. Values in italics are considered significant.

^b^ Some test measures could not be collected. Total observations in each row: 92 < *N* < 96. Medians and percentages exclude missing data.

### MPI

The MPI showed a relationship with mortality risk after the 90-day FU for older patients admitted to IMC or ICU. The mortality rate in the high-risk group tended to be higher than in the low- and medium-risk group (*p* = 0.087). When looking at the continuous score, deceased patients had a median MPI of 0.69 (IQR = 0.56–0.78), while survivors scored 0.63 (IQR = 0.50–0.69). A logistic regression showed significant MEs after adjusting for age and gender (*p* = 0.039). Subassessments revealed poorer ADL (*p* = 0.005) and IADL (*p* = 0.022) scores in deceased patients. SPMSQ (*p* = 0.004) and ESS (*p* = 0.005) also showed significantly worse results for deceased patients. In a separate evaluation of the MPI for ICU and IMC, only the result for the ICU was statistically significant (ICU *p* = 0.004 vs IMC *p* = 0.257).

### SOFA

The predictive power of the SOFA score for patients’ outcome was assessed by comparing median scores between deceased and surviving patients at the 90-day FU. The median SOFA score for deceased patients (5, IQR = 3–7) was significantly higher than for survivors (4, IQR = 2–5) (logistic regression, *p* = 0.014, Mann–Whitney U‑test, *p* = 0.040). In the SOFA subdomains, deceased patients displayed a significantly lower mean arterial pressure (*p* = 0.027) and a higher number of taken vasopressors (*p* = 0.055). As with the MPI, the separate evaluation of the SOFA score for the ICU and IMC only yielded significant results for the ICU (ICU *p* < 0.01 vs. IMC *p* = 0.238).

### Comparison of the MPI and SOFA score

**Fig. 2:**
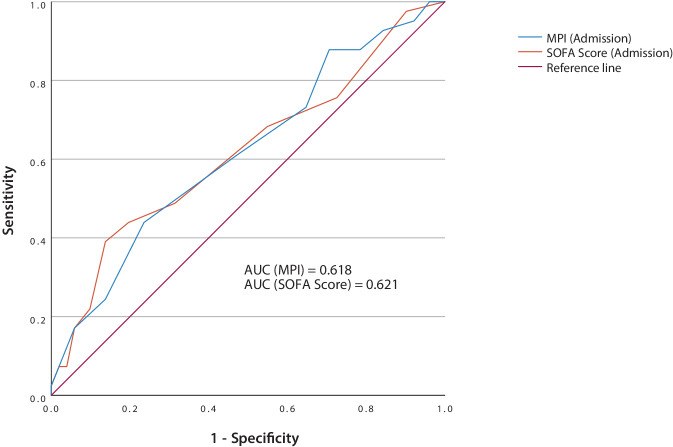
Prediction of mortality by Multidimensional Prognostic Index (MPI) and the Sequential Organ Failure Assessment (SOFA) score. *AUC* Area under the curve

To compare predictive performance, a ROC analysis, illustrated in Fig. 2, was conducted for MPI and SOFA score in predicting mortality after 90 days. The area under the curve (AUC) was 0.618 (95% confidence interval [CI] 0.502–0.733) for the MPI and 0.621 for the SOFA score (95% CI 0.507–0.735). The sensitivity was 61.0% for the MPI (cut-off = median MPI (0.688)) and 68.9% for the SOFA score (cut-off = median SOFA score (4)). The specificity was 52.9% for MPI and 45.1% for SOFA score.

### Follow-up evaluation

**Fig. 3:**
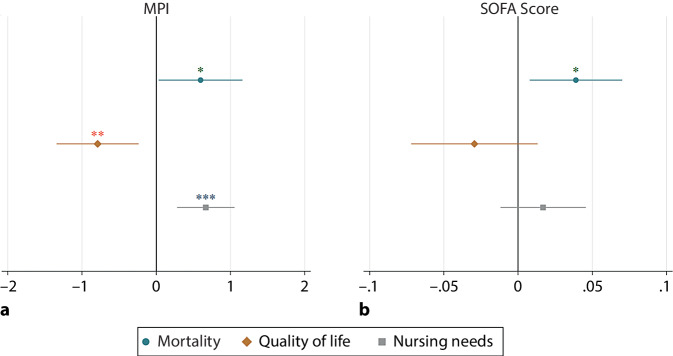
Predictive power of the Multidimensional Prognostic Index (MPI, **a**) and the Sequential Organ Failure Assessment (SOFA) score (**b**) for different patient outcomes (mortality, quality of life, and nursing needs). Each estimate is the result of a separate regression. In the case of mortality and nursing needs, estimations are based on logistic regressions, and in the case of quality of life, estimates are based on ordinary least squares (OLS) regressions. All regressions are adjusted for patient age and gender. *Points* on the horizontal axes represent the size of the marginal effects, and *whiskers* indicate 95% confidence intervals. ***p < 0.001, **p < 0.01, *p < 0.05

A higher MPI at admission showed a significant negative correlation with quality of life (*p* < 0.001, r = −0.631 at discharge, *p* = 0.005, r = −0.377 at 30-day FU; *p* = 0.004, r = −0.409 at 90-day FU) and was associated with higher nursing needs (Mann–Whitney U‑test, *p* = 0.002 at 30-day FU; *p* = 0.011 at 90-day FU) at FU. Figure 3 summarizes the predictive power of MPI and SOFA score for different patient outcomes after the 90-day FU. The figure reports coefficients from age- and gender-adjusted logistic/OLS regressions of different patient outcomes on MPI and SOFA score. As can be seen, both scores significantly predicted mortality rates, with higher scores being associated with a higher mortality risk (MPI: *p* = 0.039, SOFA score: *p* = 0.014). By contrast, only the MPI showed a significant relationship when it comes to quality of life (MPI: *p* = 0.006, SOFA score: 0.172) and nursing needs (MPI: *p* = 0.001, SOFA score: *p* = 0.248). A higher MPI was correlated with lower quality of life and a higher likelihood of the patient having nursing needs. Even though the effects went in the same direction for the SOFA score, they were not significant. In addition to MPI and SOFA score, people who survived also differed from those who died with respect to geriatric resources and syndromes. Survivors had overall more GR (*p* = 0.046)—physical (*p* = 0.005), intellectual (*p* = 0.018), and general competencies (*p* = 0.005)—than patients who were deceased at FU. When it comes to GS, survivors showed less instability (*p* = 0.039), cognitive impairment (*p* = 0.027), and delirium/incoherence (*p* = 0.046) than patients deceased at FU.

## Discussion

Due to the demographic change and the rising age of patients globally, the first AWMF (*Arbeitsgemeinschaft der wissenschaftlichen medizinischen Fachgesellschaften*) guideline was just developed to support clinical decision-making in hospitalized older patients [2]. This study aimed to assess the efficacy of the CGA-based MPI in comparison to the SOFA score for predicting mortality risk, nursing needs, and quality of life among older patients in critical care units. The investigation reveals that both instruments are comparable in terms of prognostic power for mortality. In particular the MPI showed highest accuracy in predicting not only mortality risk but also quality of life and nursing needs.

The FU assessments after 30 and 90 days showed that the MPI, but not the SOFA score was correlated with a lower quality of life and a higher probability of having nursing needs, indicative of greater prognostic complexity and healthcare needs [5, 12, 27]. This suggests that the physiological assessment captured by the SOFA score may not fully encapsulate the multidimensional aspects of health status and nursing needs among older patients in critical care settings, as captured by the MPI. In line with our findings, a study conducted in the emergency department already showed that there is a correlation between poor quality of life and a higher MPI [29].

Previous research has consistently established the MPI’s significant and sensitive association with various outcomes, including mortality, hospital stays, nursing needs, (re)hospitalization, and geriatric syndromes [8, 19, 22–24]. While affirming the existing evidence in the geriatric field, this study extends the application of the MPI to ICU and IMC. The research emphasizes that the major impact on poor outcomes is rooted in social, functional, cognitive, and nutritional aspects rather than chronological age alone [14]. Long-term health is a multifaceted concept, encompassing not only the absence of disease but also mental, social, and overall well-being [35]. Recognizing the importance of frailty and individual biological age, the MPI emerges as a suitable tool for objectively assessing frailty as a marker of biological age in the clinical setting [11, 28]. Additionally, the MPI contributes essential prognostic information and aids in clinical decision-making by classifying patients into risk groups [26, 34, 36]. The results of the study indicate that also in critical care units, a higher MPI is associated with higher mortality rates after 3 months, addressing a critical gap in intensive care decision-making.

Traditionally, the MPI has been predominantly employed for geriatric patients in internal or geriatric wards, while the SOFA is commonly utilized in intensive care units [7, 18, 24]. The study’s results demonstrate that both the MPI and SOFA score exhibit comparable performance in predicting mortality after 3 months (MPI *p* = 0.039 vs. SOFA score *p* = 0.014). The ROC analyses indicate similar sensitivity (61.0% vs. 68.9%), specificity (52.9% vs. 45.1%), and nearly identical AUC values (0.618 vs. 0.621).

While both tests effectively predict mortality, the study may initially suggest that the SOFA score is more suitable for critical care scenarios due to its shorter assessment time (approximately 10 min) compared to the MPI (approximately 30 min). However, a 2022 study introduced a short version of the MPI, called BRIEF-MPI, which was shown to be as accurate as the original MPI with an average application time of 5 min [4]. Given our study’s findings of comparable mortality risk prediction after 90 days between the MPI and SOFA score and the MPI’s superior performance in assessing quality of life and nursing needs, the MPI may be a better tool for older patients in critical care than the SOFA score.

The MPI provides valuable insights into the social and functional characteristics, quality of life, and nursing needs of older intensive care patients. The results suggest that the SOFA score is sufficient when the main interest is in understanding mortality. If, by contrast, the aim is to understand other aspects of health status and quality of life, the MPI may be more suitable. Furthermore, the comparable performance of both scores in the overall cohort is mirrored in the subgroups of ICU and IMC, although larger data collection is needed for conclusive evidence. The fact that the original MPI provides estimates of mortality risk up to 1 year [24] and, thus, exceeds the time frame of SOFA implies further potential superiority for longer FU periods. This emphasizes the importance of future research with longer FU periods to draw more differentiated conclusions.

Acknowledging limitations, the study is constrained by a single-center retrospective design, a relatively modest sample size, and observed attrition between FU assessments. While retention rates align with other studies involving multimorbid patients, findings may require replication across diverse centers with varying environmental factors. The utilization of distinct evaluators introduces potential interexaminer variations, compounded by the inability to cross-validate their assessments. Additionally, the pooling of observations from ICU and IMC, with an emphasis on IMC, introduces a potential source of bias, warranting consideration in the interpretation of results.

## Summary

- Person-centered, multidimensional prognostic evaluation and organ-based prognosis are equally valid instruments for clinical decision-making in older, critically ill patients.
- The Multidimensional Prognostic Index (MPI) is superior to the Sequential Organ Failure Assessment (SOFA) score when it comes to predicting quality of life and nursing needs (*Pflegegrad*) up to 3 months after ICU discharge.
- Findings underscore the importance of broader validation for both MPI and SOFA score, revealing a research gap in treatments for older patients in critical care settings.
- Further research is needed to determine whether MPI excels in assessing long-term prognosis for older critically ill patients, addressing a current knowledge gap.

## Data Availability

The data supporting this study’s findings are available on request from the corresponding author.

## Declarations

Ethical approval was granted by the Cologne Ethics Committee (EK19-1049_1, 18 June 2019), and the study was registered in the German Clinical Trials Registry (DRKS00016951). Informed consent was obtained from all patients or their authorized representatives.
